# Penile Microvascular Arterial Bypass Surgery: Indications, Outcomes, and Complications

**DOI:** 10.1100/tsw.2010.153

**Published:** 2010-08-17

**Authors:** Ricardo Munarriz

**Affiliations:** Department of Urology and Center for Sexual Medicine, Boston University School of Medicine, Boston, MA, United States

**Keywords:** vasculogenic erectile dysfunction, penile microarterial bypass surgery

## Abstract

Penile microarterial bypass surgery (MABS) may be the only treatment capable of restoring normal erectile function without the necessity for the chronic use of vasoactive medications or placement of a penile prosthesis. Lack of standardization in patient selection, hemodynamic evaluation, surgical technique, and limited long-term outcome data using validated instruments has resulted in this surgery being considered experimental. The members of the Erectile Dysfunction Guideline Update Panel reviewed the available MABS publications and only four of 31 manuscripts met the criteria for the Arterial Occlusive Disease Index patient. The total studied population of these four publications was 50, which was considered too small to determine if MABS is effective or not. Reported successful outcomes were 36-80 and 91% for inferior epigastric artery (IEA) to dorsal vein and IEA to dorsal artery MABS, respectively. We recently published the largest long-term outcome MABS study using validated questionnaires (71 men aged 30.5 ± 9.2 years; mean follow-up 34.5 ± 18 months). The mean pre- and postoperative total International Index of Erectile Function, erectile function domain, questions 3 and 4 scores were 35.5 ± 14.8, 13.7 ± 6.7, 2.2 ± 1.4, 2.1 ± 1.3, and 56.2 ± 16.6, 23.8 ± 6.6, 4.1 ± 1.4, and 3.9 ± 1.5, respectively. In addition, 55 and 73% of patients reported erectile function domain scores ≥26 and 21, respectively, and almost 90% of patients would recommend or undergo MABS again. In addition, changes in pre- and postoperative sexual distress scale scores and the Center for Epidemiologic Studies Depression Scale were statistically significant. More importantly, treatment satisfaction (EDITS) was very high, and 87% of patients would recommend the surgery to someone else and 88.7% reported a significant improvement in their erectile function. In patients with no vascular risk factors and pure cavernosal arterial insufficiency, MABS provides long-term improvements in erectile function, depression, and overall satisfaction.

## INTRODUCTION

Erectile dysfunction is defined as the consistent inability to obtain or maintain a penile erection satisfactory for sexual relations. Community epidemiologic studies have revealed that 52% of men aged 40–70 years have self-reported (17%), moderate (25%), and complete (10%) forms of impotence[1]. Understanding of the penile erectile physiology and pathophysiology has resulted in a variety of therapeutic modalities, which include phosphodiesterase inhibitors (PDEs), intracavernosal therapy, vacuum constrictive devices, penile prostheses, and arterial revascularization procedures.

Penile microarterial bypass surgery (MABS) was first described by Michal et al. in 1972[2] and is considered by many as a breakthrough in the treatment of erectile dysfunction, since it is the only treatment modality capable of restoring normal erectile function without the necessity of external mechanical devices, chronic use of vasoactive medications, or surgical placement of internal penile prosthetic devices. The overall goal of penile revascularization surgery is to bypass obstructive arterial lesions in the hypogastric-cavernous arterial bed. The specific objective of the surgery is to increase the cavernosal arterial perfusion pressure and blood inflow in patients with vasculogenic erectile dysfunction secondary to pure arterial insufficiency[3].

Nevertheless, lack of standardization in patient selection, hemodynamic evaluation, and surgical technique as well as limited long-term outcome data using validated instruments has resulted in this surgery being considered by many surgeons as an experimental procedure. However, the members of the Erectile Dysfunction Guideline Update Panel gave the following recommendation: “Arterial reconstructive surgery is a treatment option only in healthy individuals with recently acquired erectile dysfunction secondary to a focal arterial occlusion and in the absence of any evidence of generalized vascular disease”[4]. In addition, poor reimbursements, long surgical time, and the need for microsurgical expertise have led to infrequent penile revascularization procedures.

## HISTORY

Michal et al. reported the first MABS in 1972[2]. Michal and his collaborators directly anastomosed the inferior epigastric artery (IEA) to the corpus cavernosum (Michal I), which resulted in flow rates of more than 100 mL/min and intraoperative erections. Unfortunately, this technique had an almost 100% anastomotic stenosis and unsatisfactory success rates. They subsequently, anastomosed the IEA (end-to-side) with the dorsal penile artery (DPA) with a 56% success rate (Michal II)[5].

Virag et al. reported a 49% success rate and an additional 20% improvement by anastomosing the IEA to the deep dorsal vein, which in a retrograde fashion increased penile perfusion[6]. Furlow and Fisher also performed arterialization procedures of the dorsal vein with ligation of the circumflex branches to avoid glanular hyperemia, with success rates of 62%[7]. Hauri reported a complicated side-to-side anastomosis between the dorsal artery and vein covered by a spatulated IEA that resulted in success rates of 80%[8].

Current MABS techniques consist of an anastomosis between the IEA to the dorsal vein or artery. Artery-to-vein MABS are technically easier than artery-to-artery, but in our opinion, they are more likely to fail over time for several reasons. First, the dorsal vein has valves that most likely impair penile reperfusion and may be associated with anastomotic thrombosis. The use of a valvulotome may improve retrograde reperfusion to the corpora, but may also cause endothelial injury, which may activate the intrinsic pathway of the clotting system, leading to early thrombosis and failures. Finally, artery-to-artery MABS eliminates the possibility of penile hyperemia. Unfortunately, our opinion regarding which technique is better is not supported by the data. However, our outcome data seem to be better than artery-to-vein reports by other authors and are consistent with that from Jarow and DeFranzo’s 1996 publication[9]. In consequence, we favor IEA to dorsal artery MABS.

## PENILE VASCULAR ANATOMY

The penis consists of three cylindrical erectile bodies: the paired corpora cavernosa, the key structures mediating penile erection, and the corpus spongiosum. Each corpus cavernosum has a thick fibrous sheath, the tunica albuginea, which surrounds the whole cavernosal erectile tissue. Bulbospongiosus muscles surround the corpus spongiosum in the bulbar region and facilitate ejaculation through rhythmic contractions. A thick elastic layer, the Buck's fascia, surrounds and is firmly attached to the tunica albuginea. Blood vessels and nerves run between the Buck's fascia and the tunica albuginea.

The blood supply to each corpus cavernosum is derived mainly from the internal pudendal artery, which gives three terminal branches: the bulbourethral, the scrotal, and the common penile artery, which divides into the forsal and cavernosal arteries. The internal pudendal artery enters the perineum at the level of Alcock's canal, being vulnerable to compression injuries, such as may occur during bicycle riding[10,11]. It has recently been demonstrated that the dorsal artery interconnects with the cavernosal artery, allowing arterial bypass surgery in the case of cavernosal artery obstruction. Frequently, accessory pudendal arteries provide additional blood to the corpora cavernosa and may have a critical role in men who undergo pelvic surgery.

Blood leaves the penis via three venous systems: superficial, intermediate, and deep. The superficial system (superficial dorsal veins) consists of small venous channels in the subcutaneous layer draining the skin and subcutaneous tissue above the Buck's fascia. The intermediate system (deep dorsal vein and circumflex veins) drains blood from the glans, corpus spongiosum, and the distal two-thirds of the corpora. Venous blood from the corporal sinusoids drains initially into tiny subtunical venules, which merge into emissary veins, penetrating the tunica albuginea, and empty into the deep dorsal vein, directly or via the circumflex veins. The deep system (cavernous and crural veins) is the main drainage system of the corpora cavernosa and a main source of leakage in venogenic impotence.

The sponge-like corpus cavernosum consists of bundles of smooth muscle cells embedded in a matrix of connective tissue and fibroblasts, forming trabeculated structures that define a series of blood-filled lacunar spaces lined with endothelium. In the flaccid state, the trabecular smooth muscle and the central cavernosal and branching helicine arteries within each cavernosal body are constricted. During the transition to the erect state, sacral parasympathetic stimulation causes the dilation of the cavernosal and helicine arteries, enabling a higher rate of blood flow into the penis. Concomitant relaxation of the trabecular smooth muscle greatly increases the compliance of the cavernosal bodies and allows the lacunar spaces to expand and accommodate the enhanced blood flow. Additionally, the outflow of blood must be reduced in order to achieve and maintain full tumescence and rigidity. The restriction of venous outflow from the cavernosal bodies is accomplished by elongation and compression of subtunical venules between the expanding trabecular structures and the tunica albuginea. This process is known as the veno-occlusive mechanism.

## PATIENT SELECTION

There is no consensus in the criteria for patient selection. However, the members of the Erectile Dysfunction Guideline Update Panel provided a definition of the Arterial Occlusive Disease Index patient. The Panel assumed that the patient who is likely to benefit from arterial reconstructive surgery is an otherwise healthy man, 55 years old or younger, with recently acquired erectile dysfunction due to focal arterial occlusive disease. The reason for including the criteria of recently acquired onset and the absence of other risk factors, such as smoking, diabetes, or others, in this definition was to eliminate patients with either diffuse vascular disease or cavernous myopathy due to chronic ischemia[4].

At our center, patients undergo a psychological, medical, and hormonal evaluation, and must meet the following inclusion and exclusion criteria to be considered candidates for MABS:

1. Age <55 years
2. Absence of vascular risk factors (diabetes, hypertension, tobacco use, hypercholesterolemia)

3. No evidence of neurologic erectile dysfunction (e.g., multiple sclerosis, pelvic surgery, lumbosacral radiculopathies, etc.)
4. No hormonal abnormalities
5. No evidence of active psychiatric disorders (severe depression, bipolar disease, schizophrenia)
6. No evidence of Peyronie's disease
7. Absence of premature ejaculation
8. Report of acute or chronic perineal/pelvic trauma
9. No evidence of corporo-occlusive dysfunction by duplex Doppler ultrasound and cavernosometry
10. Focal occlusive disease of the common penile or cavernosal arteries documented by penile duplex Doppler ultrasound, cavernosometry, and selective internal pudendal arteriography

## HEMODYNAMIC EVALUATION

We recommend a screening penile duplex Doppler ultrasound with redosing if necessary[12]. Peak systolic velocities (PSV) <25 cm/sec are considered diagnostic, and velocities between 25 and 30 cm/sec borderline for arterial insufficiency**.** However, Chung and colleagues reported that PSV are age dependent, so in cases of borderline PSV in young men, a cavernosometry/cavernosography may be more accurate[13]. In addition, end diastolic velocities and resistive index should be consistent with normal corporo-occlusive function. We routinely performed cavernosometry to further document arterial occlusive disease and normal corporo-occlusive function. Flows to maintain <5 cc/min and pressure decays <45 mm of Hg over 30 sec rules out the possibility of venous leak. In addition, arterial gradients >20 mm of Hg between the brachial and cavernosal arteries confirm the diagnosis of arterial insufficiency. Finally, a selective internal pudendal arteriogram will confirm the presence and localization of a focal occlusion and provides invaluable information on the donor (IEA) and recipient vessel (dorsal artery) (e.g., size, length, branches)[14].

## SURGICAL TECHNIQUE

From a technical standpoint, the operation is divided into three stages: dorsal artery dissection, IEA harvesting, and microsurgical anastomosis[2].

1. ***Dorsal Artery Dissection:*** A 5-cm semi-lunar scrotal incision 2 cm below the penoscrotal junction is made in the side opposite to the planned abdominal incision for IEA harvesting. With the penis stretched, finger blunt dissection along Buck's fascia towards the glands allows for inversion of the penis. The fundiform ligament is identified and preserved to minimize penile shortening. The selected dorsal artery is isolated and mobilized proximally, avoiding injury of the dorsal nerves. Temporary scrotal closure is performed with staples (Fig. 1).
2. ***Harvesting of the IEA***: A 5-cm transverse incision three-quarters distance between umbilicus and pubis is created with a scalpel. Dissection is carried down through Scarpa's fascia using the bovie, the rectus fascia is divided vertically, and the rectus muscle mobilized medially. The IEA is identified and mobilized from its origin at the level of the common external iliac artery to the umbilicus (Fig. 2). If arterial branches are found, they are controlled with bipolar cautery and divided. During the mobilization of the IEA, papaverine is utilized to prevent vasospasm. The distal end of the IEA is clipped near the umbilicus and divided. Subsequently, the scrotal staples are removed and a Schnitt clamp is utilized to transfer the IEA to the dorsal aspect of the penis through the external inguinal ring, which was previously bluntly dilated. The abdomen is closed in a multilayer fashion using a running technique with 0 polyglycolic acid suture for the rectus fascia, 2-0 for Scarpa's, and a 4-0 monocryl for the skin.

3. ***Microvascular Anastomosis:*** The penis is reinverted and the operating microscope brought to the surgical field. A Scott retractor is utilized to accommodate the penis for microsurgery. The dorsal artery is mobilized and divided in a proximal location on the penile shaft. The proximal end is cauterized using the bipolar cautery. Aneurismal clips are place on the dorsal artery and IEA. The adventitia of the distal end of the IEA and proximal dorsal artery is sharply excised with

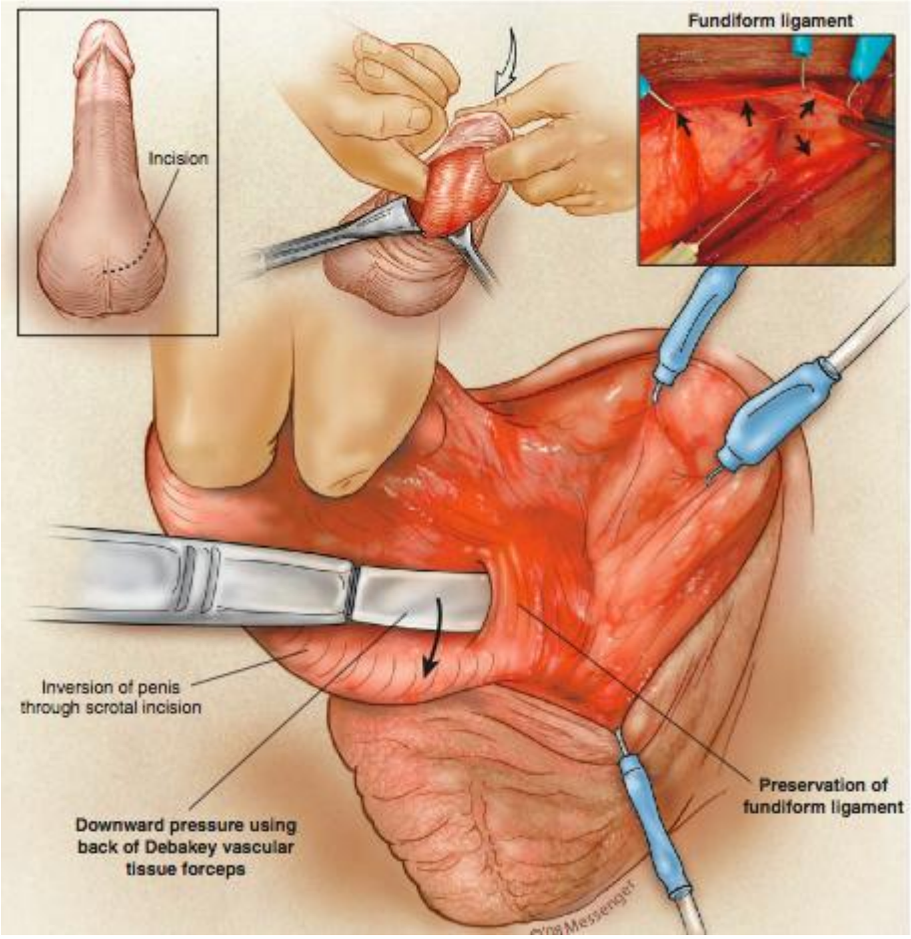

**FIGURE 1.** Dorsal artery dissection. (From Goldstein, I., Bastuba, M., Lurie, A., and Lubisich, J. [2008] Penile revascularization. J. Sex. Med. 5(9), 2018-2021. With permission.)

microscissors to prevent thrombosis of the anastomosis. A microsurgical anastomosis is performed using a simple interrupted technique with 10-0 Nylon stitches (Fig. 3). The dorsal aneurismal clip is removed and back blood flow is observed, documenting anastomotic patency. The IEA aneurismal clip is removed and if there is no anastomotic leak, the penis is placed back on its normal anatomical position, the Dartos closed with a running 2-0 polyglycolic acid suture, and the skin with a 4-0 polyglycolic acid suture. Patency of the anastomosis is further confirmed by Doppler ultrasound.

## RESULTS

In 2005, the members of the Erectile Dysfunction Guideline Update Panel reviewed the available MABS publications and only four of 31 manuscripts met the criteria for the Arterial Occlusive Disease Index patient[4]. The total studied population of these four publications was 50, which was considered too small to determine if MABS is effective or not. Reported successful outcomes were 36–80 and 91% for IEA to dorsal vein and IEA to dorsal artery MABS, respectively (Table 1). We recently published the largest long-term outcome MABS study using validated instruments (71 men, aged 30.5 ± 9.2 years, mean follow-up 34.5 ± 18 months)[13]. The mean pre- and postoperative total International Index of Erectile Function, erectile function domain, questions 3 and 4 scores without the use of PDE5 type inhibitors were

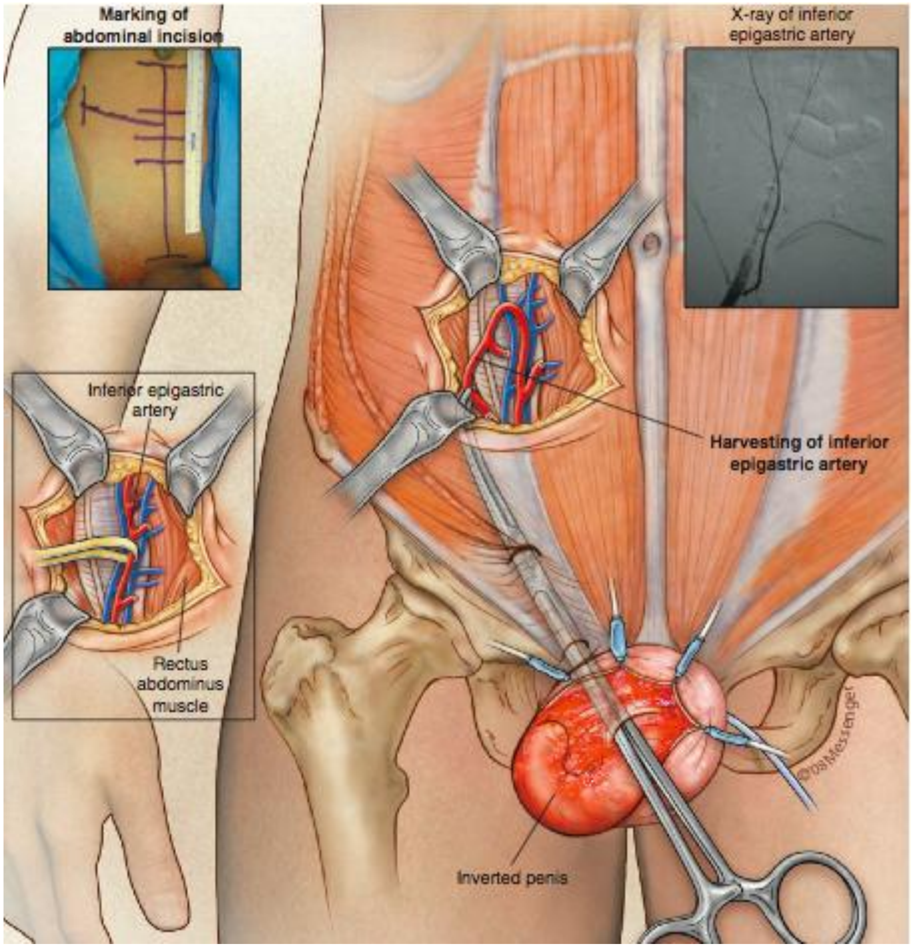

**FIGURE 2.** Harvesting of the IEA. (From Goldstein, I., Bastuba, M., Lurie, A., and Lubisich, J. [2008] Penile revascularization. J. Sex. Med. 5(9), 2018-2021. With permission.)

35.5 ± 14.8, 13.7 ± 6.7, 2.2 ± 1.4, 2.1 ± 1.3, and 56.2 ± 16.6, 23.8 ± 6.6, 4.1 ± 1.4, and 3.9 ± 1.5, respectively. In addition, 55 and 73% of patients scored erectile function domain scores ≥26 and 21 without the need of PDE5 inhibitors, and almost 90% of patients would recommend or undergo MABS again. Preoperative penile rigidity assessed by an analog scale with and without PDEs was 41 and 77%, respectively, and 71 and 90.8% postoperatively. In addition, changes in pre- and postoperative sexual distress scale scores (SDS 37.7 ± 11.0 and 17.5 ± 12.5) and the Center for Epidemiologic Studies Depression Scale (CES-D 42.0 ± 10.0 and 33.7 ± 6.1) were statistically significant. More importantly, treatment satisfaction (EDITS) was very high, and 87% of patients would recommend the surgery to someone else and 88.7% reported a significant improvement in their erectile function (Table 2).

How many were functional without use of PDE5 inhibitors?? The above paragraph clearly states that 55 and 73% of patients had normal erections (erectile function domain ≥26 or 21 without the need of PDEs).

## COMPLICATIONS

Short- and long-term complications are not uncommon. However, patient satisfaction continues to be high despite them. We reported a complication rate of wound infection, inguinal hernias, loss of penile length,

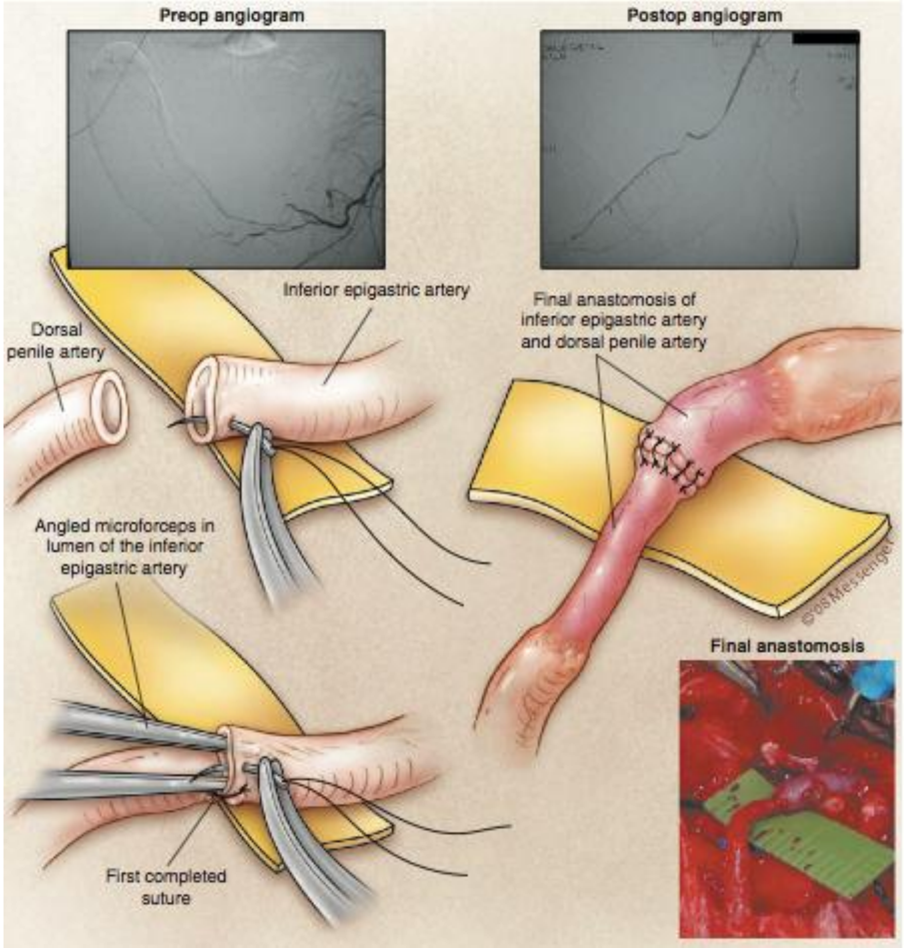

**FIGURE 3.** Microvascular anastomosis. (From Goldstein, I., Bastuba, M., Lurie, A., and Lubisich, J. [2008] Penile revascularization. J. Sex. Med. 5(9), 2018-2021. With permission.)

**TABLE 1**
**Prior Published Reports on Outcomes of Penile Revascularization that Met the Criteria for the Arterial Occlusive Disease Index Patient[4]**

| Report | No. of Patients | Follow-Up (months) | Success Rate | Success Criteria |
|---|---|---|---|---|
| Grasso (1992) | 22 | 12 | 68% w/ NPT; 36% Doppler | NPT, Doppler |
| De Palma (1995) | 11 | 12–48 | 60% | Doppler |
| Jarow (1996) | 11 | 12–84 | 91% | Doppler |
| Ang (1997) | 6 | 8–37 | 66% | NPT, Doppler |

and decreased penile sensitivity of 2.8, 2.8, 28, and 24.7%, respectively. We believe that the change in penile sensation is due to a denervation injury during surgery despite the use of 10× magnification. However, postoperative orgasmic domain scores in these patients show an improvement in orgasmic function, which could only be explained by a significant improvement of their overall sexual function and, at the same time, rules out the possibility of significant dorsal neuropathy.

**TABLE 2**
**MABS: Long-Term Outcomes Using Validated Instruments[15]**

| N = 71 | Preop | Postop |
|---|---|---|
| IIEF | | |
| Total score | 35.5 ± 14.8 | 56.2 ± 16.6 |
| EF domain of IIEF | 13.7 ± 6.7 | 23.8 ± 6.6 |
| Question 3 | 2.2 ± 1.4 | 4.1 ± 1.4 |
| Question 4 | 2.1 ± 1.3 | 3.9 ± 1.5 |
| Penile rigidity (%) | | |
| With PDEs | 41 | 71 |
| Without PDEs | 77 | 90 |
| SDS | 37.7 ± 11.0 | 17.5 ± 12.5 |
| CES-D | 42.0 ± 10.0 | 33.7 ± 6.1 |
| Edits | | 72–81% |

Penile shortening is a classic patient self-reported complication of MABS. Preservation of the fundiform ligament has anecdotally minimized this complication. Our series documents a penile shortening complication rate of 28% of patients. However, 87.7% of patients would recommend or have surgery again, which suggests that the loss of penile length is not critical for this group of well-selected men. We think that the lost penile length is due to fibrosis/scarring of the fundiform ligament and not to vascular factors (e.g., anastomotic tension).

## CONCLUSIONS

MABS is the only treatment modality capable of restoring normal erectile function without the necessity of external mechanical devices, chronic use of vasoactive medications, or surgical placement of penile prostheses. Lack of standardization in patient selection, hemodynamic evaluation, and surgical technique, as well as limited long-term outcome data using validated instruments, has resulted in this surgery being considered by many surgeons as an experimental procedure.
